# Motmot, an open-source toolkit for realtime video acquisition and analysis

**DOI:** 10.1186/1751-0473-4-5

**Published:** 2009-07-22

**Authors:** Andrew D Straw, Michael H Dickinson

**Affiliations:** 1 Bioengineering, California Institute of Technology, Mailcode 138-78, Pasadena, CA 91125, USA

## Abstract

**Background:**

Video cameras sense passively from a distance, offer a rich information stream, and provide intuitively meaningful raw data. Camera-based imaging has thus proven critical for many advances in neuroscience and biology, with applications ranging from cellular imaging of fluorescent dyes to tracking of whole-animal behavior at ecologically relevant spatial scales.

**Results:**

Here we present 'Motmot': an open-source software suite for acquiring, displaying, saving, and analyzing digital video in real-time. At the highest level, Motmot is written in the Python computer language. The large amounts of data produced by digital cameras are handled by low-level, optimized functions, usually written in C. This high-level/low-level partitioning and use of select external libraries allow Motmot, with only modest complexity, to perform well as a core technology for many high-performance imaging tasks. In its current form, Motmot allows for: (1) image acquisition from a variety of camera interfaces (package motmot.cam_iface), (2) the display of these images with minimal latency and computer resources using wxPython and OpenGL (package motmot.wxglvideo), (3) saving images with no compression in a single-pass, low-CPU-use format (package motmot.FlyMovieFormat), (4) a pluggable framework for custom analysis of images in realtime and (5) firmware for an inexpensive USB device to synchronize image acquisition across multiple cameras, with analog input, or with other hardware devices (package motmot.fview_ext_trig). These capabilities are brought together in a graphical user interface, called 'FView', allowing an end user to easily view and save digital video without writing any code. One plugin for FView, 'FlyTrax', which tracks the movement of fruit flies in real-time, is included with Motmot, and is described to illustrate the capabilities of FView.

**Conclusion:**

Motmot enables realtime image processing and display using the Python computer language. In addition to the provided complete applications, the architecture allows the user to write relatively simple plugins, which can accomplish a variety of computer vision tasks and be integrated within larger software systems. The software is available at

## Background

The combination of video cameras and realtime image analysis offers the experimenter a sophisticated, non-invasive toolset to observe and automatically interact with dynamic processes, such as sensory-motor behaviors of animals. Real time image analysis is now more feasible as digital cameras have become inexpensive and computers capable of high performance computation become commonplace.

**Figure 1:**
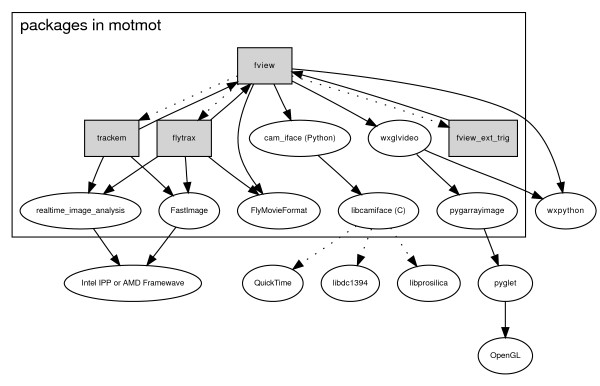
**Relationships of packages inside and outside motmot**. Motmot is a collection of related packages that allow for acquisition, display, and analysis of realtime image streams from uncompressed digital cameras. The packages that comprise motmot are within the large box. Shaded boxes are GUI applications (fview) and plugins (flytrax, trackem and motmot.fview_ext_trig) that end-users can utilize directly. Arrows represent a dependency such that the node at the head of the arrow depends on the node at the tail. Dotted lines represent an optional (plugin) relationship. Not listed are the Python language itself and numpy numerical processing library, which are dependencies of nearly all motmot packages.

We describe here 'Motmot', a set of software packages designed to allow use of video technology with particular emphasis on neuroscience applications (see Figure 1 and Table 1). Of paramount importance in such applications is the ability to integrate the video system with other experimental components with maximal temporal certainty. For example, it may be critical to know the location or orientation of an experimental subject at the moment of stimulus onset and track movement with high temporal precision and low latency. 'Virtual reality' video displays, and the psychophysics experiments performed using them, are contingent on low latency tracking. Humans are capable of perceiving visual motor latencies less than 20 msec [1,2], and it is reasonable to assume animals with faster visual and motor systems may be sensitive to even shorter latencies. In other experiments, correlation of electrophysiological recordings with animal movement might be required. In this case, the precise (sub-millisecond) relative timing of spikes and limb movement may be desired. The Motmot software was designed to facilitate image acquisition and analysis with these types of requirements. With an inexpensive USB device called 'CamTrig', integration with other experimental components can be achieved with precise temporal synchronization. The hardware for CamTrig may be purchased commercially and the firmware is included with Motmot.

**Table 1:** Motmot components

**High-level GUI Application**	
motmot.fview	Application with plugin architecture to enable writing new realtime analyses by creating one's own process_frame() function.

**Core camera infrastructure**	

libcamiface	Camera interface C API

motmot.cam_iface	Python wrapper of libcamiface

CamTrig	Firmware for the a USB device for precise temporal synchronization and analog input

**Core display infrastructure**	

motmot.wxglvideo	wxPython OpenGL interface for video

pygarrayimage	Transfer Python objects supporting the array interface to OpenGL textures

motmot.wxvideo	wxPython interface for video

motmot.imops	Python extension module to manipulate image codings

**Analysis infrastructure**	

motmot.FastImage	Pyrex based wrapper of Intel's Integrated Performance Primitives (IPP) or AMD Framewave Library

motmot.FlyMovieFormat	Code for manipulating .fmf movies. Includes Python (read/write) and MATLAB^® ^(read-only) bindings.

motmot.realtime_image_analysis	Implements background subtraction and 2D feature extraction using FastImage

**FView plugins**	

motmot.fview_ext_trig	software for the CamTrig USB Device

motmot.flytrax	FView plugin for tracking 2D points in realtime and saving data and small images. (Used in [6-8].)

motmot.fview_PLUGIN_TEMPLATE	blank fview plugin to use as template for new plugins

motmot.fview_c_callback	example fview plugin that calls pure C code

motmot.fview_live_histogram	example fview plugin that calls pure Python code

motmot.trackem	multiple point realtime tracker (used in [12])

Motmot consists of several modular components. Brief descriptions are given here, and the relationships between the components are shown in Figure 1.

At least one other open-source package with similar capabilities is available [3], although it is focused primarily on microscopy applications, whereas the emphasis of Motmot is on behavioral applications with realtime image analysis plugins.

This paper describes the important concepts behind Motmot. These include an overview of temporal synchronization issues, a discussion of the use of Python for realtime computing tasks, and a brief description of the primary software components of Motmot, which are available for download from . Complete instructions for downloading and installation are available at the website.

## Synchronizing multiple clocks

Fundamental to Motmot is the ability to reconstruct what happened when. This is often difficult with computer equipment because different devices each have their own clocks and therefore (potentially) different numbers to describe a single instant. Furthermore, because communication between devices takes time, it may not be trivial to estimate differences between clocks.

One example of the experimental possibilities available if such challenges are overcome is the ability to trigger an event to happen at a specified number of milliseconds after a change in the video image – even with variable latencies in video image analysis, clock synchronization makes this possible. Another possibility is the ability to track animals with multiple cameras using multiple computers and to compare simultaneous images in order to estimate 3D position. Without precise temporal alignment, the 3D reconstruction process would be subject to measurement errors of the magnitude of the distance the animal traveled over the duration of the temporal alignment error.

Motmot solves these problems by providing accurate estimates of the time of external events with reference to the computer's own clock. With this information known, accurate temporal correspondences may be used for analysis purposes. Furthermore, because accurate timing information is computed online, experimental designs in which timing is critical are also possible (for example, triggering an event with specified latency as described above). Finally, the clocks of multiple computers may be brought into precise alignment (within one or two microseconds over a typical local area network) using implementations of the IEEE-1588 Precise Time Protocol such as PTPd [4]. By coordinating the clocks of multiple computers, experiments requiring more computing power than is available within a single computer are possible. For example, using Motmot, we have implemented an eleven camera realtime free-flight fly tracking system, described below in the 'External applications' section.

### The camera and the computer

In the simplest case, it is desirable to know when an image was acquired in the computer's own time base without any additional equipment. To allow this, the Motmot camera interface 'libcamiface' queries the camera drivers for a timestamp associated with each frame. Some camera drivers return a timestamp in units of the computer's own clock (libdc1394, for example). In this case, a correlation with images and other events recorded on the computer can be made. In other systems, the timestamp returned by the camera driver is not in the same units as the computer's clock (for example, with the Prosilica GigE SDK) and no such correlation can easily be made, leaving it difficult to determine, beyond an approximation on the order of ten milliseconds or more, exactly when an image was acquired.

### CamTrig USB device

With an additional piece of equipment, the CamTrig USB device, any camera whose image acquisition can be triggered on arrival of an external voltage pulse can be correlated with high precision (sub-millisecond) to the computer's clock. The CamTrig USB device is an inexpensive ($30) USB-based microcontroller device, the Atmel USBKEY, loaded with custom firmware provided as part of Motmot. This firmware may be directly installed as-is. Alternatively, the source code (based on the LUFA library) may be modified and compiled with the free GCC-AVR cross compiler.

**Figure 2:**
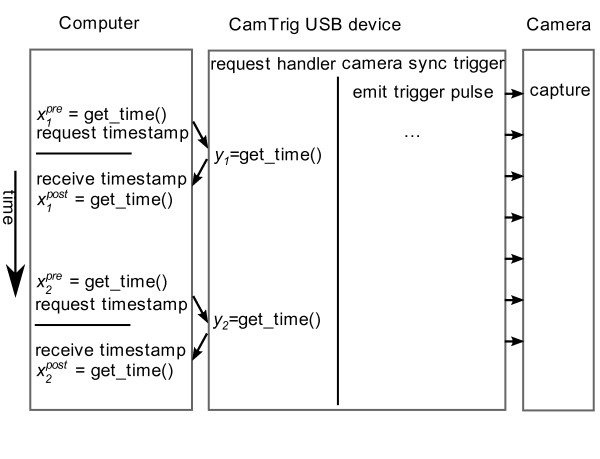
**Overview of CamTrig USB operation for synchronizing multiple clocks**. As described in the text, the CamTrig USB device allows the host computer to accurately estimate the timing of external events. The clock onboard the CamTrig USB device is a crystal oscillator driving a counter/timer to emit trigger pulses at very regular intervals. USB requests allow the host computer to build an accurate model of the time of events on the CamTrig device in its own time frame of reference.

Amongst other functions (described below), the CamTrig firmware emits regularly timed trigger pulses at a specified frequency to begin image acquisition (see Figure 2). The motmot.fview_ext_trig package queries the device every few seconds over USB about the value of its clock, and thus its frame counter. With these measurements, the clock onboard the CamTrig device is modeled relative to the computer's internal clock using a simple linear model

(1)

where *A *is the gain between the two clocks and *b *is the offset.

The motmot.fview_ext_trig package estimates the model coefficients by sampling the relevant values (shown in Figure 2). First, an individual sample of the CamTrig's time *y*_*n *_is made. This sample is estimated to have occured at time *x*_*n *_on the host computer using a symmetric delay assumption

(2)

Once two or more samples are acquired, a linear least squares fitting routine is applied, and *A *and *b *are estimated. Thus, from any value of host clock *x*, the corresponding CamTrig time *y *can be determined and vice versa. With more than two samples, the fit is overdetermined and the effect of additive noise is reduced. Additionally, one class of large measurment errors is automatically rejected by ignoring values of  exceeding a threshold. Finally, by performing the linear least squares fit on acquisition of each new sample using only the most recent 100 samples, the clocks' relationship is allowed to slowly drift over time, accounting for slow non-linearities in the clocks' own rates.

With regularly timed trigger pulses and a model of the time of those pulses as a function of the computer's internal clock, a precise reconstruction of the time of the trigger pulse can be made. With camera-specific information about the latency from trigger pulse to image acquisition onset (usually negligibly small – on the order of nanoseconds) and camera-driver specific information about the duration of the image integration time, very accurate estimates of image acquisition start and stop may be made for each and every image.

### Additional capabilities of the CamTrig USB device

The CamTrig USB Device provides analog input and digital input/output capabilities. As described above, the clock of the CamTrig USB device is modeled with high accuracy and precision by the motmot.fview_ext_trig host software. By taking advantage of this, analog voltage samples are acquired and related precisely to the temporal model. The resulting temporal synchronization of analog input with the computer's onboard clock may be a particularly important capability of Motmot for some applications. In electrophysiological experiments, for example, it may be useful to know the precise timing of spikes recorded in an analog voltage trace relative to images acquired of animal movements. Given the relatively low analog sampling performance of the CamTrig USB Device (10-bit analog-to-digital converter, 0.0 to 3.3 volt range, up to 9.6 KHz sample rate), this device is not ideal for recording intracellular or extracellular signals directly, but is useful for recording spike times or synchronizing with other data acquisition components.

## Realtime computing in mainstream operating systems

An animal normally operates in 'closed loop', whereby its own movements cause sensory input to change. By interposing between motor output and sensory feedback, novel experimental designs are possible, such as a 'virtual open loop' condition [5], in which visual feedback from an animal's own movement is canceled by way of a computer-updated display such that objects appear to remain stationary despite the animal moving to a new location.

Computer programs executing within such a feedback loop must operate in realtime, computing results immediately after being given input. Of course, the time scales required depend on the specific circumstances, but to create virtual-reality simulations, very short latencies are desired. Human observers, for example, are sensitive to latencies as low as 15–16 milliseconds [1,2]. In walking *Drosophila *at luminances less than 60 cd/m^2^, no statistically significant differences were found at up to 200 milliseconds of latency in one measure of visually elicited turning behavior [5]. (It should be noted, however, that the experiment was not designed to detect whether flies could discriminate the two conditions but rather to show that the responses measured in virtual open loop with an apparatus with an update rate of 5 Hz were similar to those measured in true open loop.)

This section describes the difficulties associated with achieving such low latencies. After considering these issues, we implemented Motmot to run on mainstream operating systems (Linux, Windows, and Mac OS X) to allow use of a wide variety of data acquisition devices, stimulus output devices and online analysis routines. Additionally, we chose to implement the system primarily in Python to take advantage of the rapid prototyping and wide range of libraries available with this language. As described in this section, the cost of these choices is that Motmot cannot guarantee bounded latency, and the 'Latency measurements' section below quantifies the resulting performance of the system.

*Hard realtime *computer systems are defined to have a bounded maximum latency between when a task is scheduled to happen and when it actually happens. Due to the preemptive multitasking nature of today's mainstream operating systems, it is difficult or impossible to achieve hard realtime performance within a normal (user space, as opposed to kernel space) process. In particular, the kernel may preempt a time-critical process with another process, and there may be no mechanism to ensure a process is run at a certain time or immediately upon a certain event. *Soft realtime *systems have no guaranteed maximum latency, but seek to process data with a minimum of latency without any absolute performance guarantee. Computer games, for example, are soft realtime systems, and they illustrate that soft realtime feedback is sufficient to implement many interesting behavioral tasks with a computer in the sensory-motor feedback loop.

The use of a soft realtime operating system means that no bound on latency can be guaranteed. Furthermore, even if using a hard realtime operating system, it is difficult to write complex software with deterministic latency. For example, automatic 'garbage collection', a feature of several computer languages, including Python, is used to free unused memory, but this feature places indeterminacy in the execution time of a piece of code. Therefore, to implement a hard realtime application, all critical components must have deterministic latency, making language features such as garbage collection unsuitable. For some computer program functions, such as CPU interrupt service routines written in C, ensuring a bounded latency may be no problem, while for graphical displays, there may be no possibility to ensure that use of an API, such as OpenGL, does not itself introduce non-deterministic latencies. Thus, the challenge of creating a hard realtime application is formidable, and Motmot does not attempt to solve these problems. To summarize, by using a mainstream operating system, Motmot cannot guarantee bounded latency. By choosing Python as the primary implementation language, Motmot has additional indeterminacy in system latency. Thus, the total latency may be greater due to use of Python, but considered within the context of the variable latency associated with using a mainstream operating system, we viewed this as a minor quantitative effect rather than as a problematic qualitative change. The following section quantifies these issues.

## Latency measurements

Latency in a realtime camera and computer system arises from several sources. The lower bound is set by the integration time of the sensor. To that is added the duration of readout and transfer of data from the camera over the interface (such as FireWire or gigabit ethernet), and from there into the computer's main RAM. Once in RAM, a program analyzes the image and performs some action based on the outcome of these computations. Additionally, other factors that are indeterminate from the program's perspective may slow things down further (see the "Realtime computing in mainstream operating systems" section, above). We did not attempt to measure each of these components individually, because it is their cumulative sum that determines the latency of feedback and is therefore of primary interest in building realtime system. From the total latency, we can subtract the known image integration time and estimates of readout and transfer duration based on technical data from camera manufacturers. The remainder is attributable to the operating system and Motmot.

**Figure 3:**
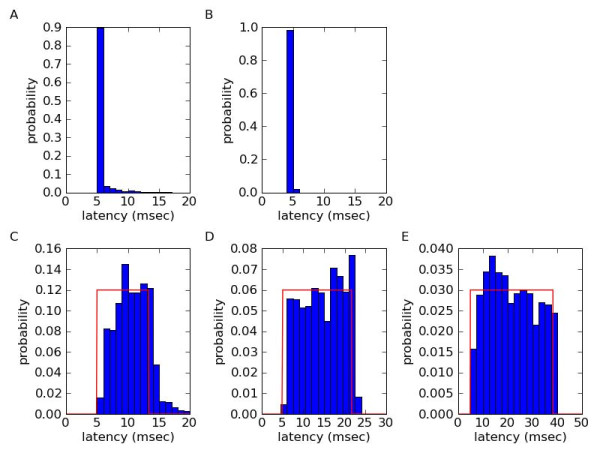
**Latency measurements using Motmot**. Latency measurements show that latency with the motmot software is close to the theoretical minimum possible given a particular camera interface. A) Latency measured as time of arrival of an image after a single trigger pulse was delivered to a camera. The camera (Prosilica GE-680) was set to 10 microsecond shutter integration interval and is expected to have a 5 millisecond transfer time to the computer. B) Latency estimated by correlating frame trigger time with computer clock time using motmot.fview_ext_trig. C) Latency measured by pulsing an LED at random times and calculating the delay until the luminance change was measured on the computer (blue histogram). The external trigger pulse given at 120 frames per second. Note that latency in this case includes the effects of the variable delay between the onset of the LED and the image acquisition as described in the 'Latency measurements' section. The red line is the theoretically predicted uniform distribution, with equal probability from 5 msec to 5 msec plus the inter-frame interval. D) as in C, but with external trigger pulses at 60 frames per second. E) as in C, but with external trigger pulses at 30 frames per second.

We measured latency in three ways (Figure 3). All three methods are included with the motmot.fview_ext_trig software package in the examples directory and may be performed on any camera that is triggered via an external trigger input via the CamTrig device.

The first method, shown in Figure 3A, is the most direct but is infeasible during ongoing image acquisition. A single image trigger pulse is given at a time measured by the host computer, and the time of arrival of the image is measured. The difference between these two time values is the acquisition latency. Because acquisition would not normally involve the host computer generating trigger pulses itself and because the pulse generation command itself may take time, this measurement is an upper bound for image transfer latency. In Figure 3A, we performed this measurement on a Prosilica GE-680 camera (Prosilica, Canada) using Prosilica GigE SDK v1.18 and Ubuntu Linux 8.04 i386 on an Acer Aspire L310 computer. Camera integration time ('shutter time') was set to 10 microseconds. The median latency in this case was about 5 milliseconds.

The second experiment to measure latency, shown in Figure 3B, involves recording the arrival time of each frame in the computer and comparing the difference from the time of the trigger pulse. This trigger pulse time is estimated using the computer's model of the clock onboard the CamTrig USB device, as described above and illustrated in Figure 2. This measurement method is suitable for latency measurements during ongoing recordings, and in the results shown, gives nearly identical results to the first method.

The third way to measure latency, shown in Figures 3C–E, involves flashing an LED in the field of view of the camera and is thus conceptually similar to the first method, but latency is measured by analyzing luminance in an ongoing image stream rather than arrival of an image. An additional complication in this scenario, however, is that images are being acquired at a regular rate whereas the LED may be illuminated at any moment relative to the image acquisition cycle. Thus, if the LED is illuminated immediately after integration ends, an almost full cycle must pass before another image is acquired. Thus, latency in this case can range from near the theoretical minimum, if the LED was illuminated simultaneously with the trigger pulse, to near a maximum determined by the reciprocal of the frame rate plus the image transfer time. For example, at a 120 Hz frame rate, with a minimum transfer latency of 5 msec, the expected latencies are uniformly distributed over the interval 5–13.3 milliseconds (13.3 = 5 + 8.3, where 8.3 msec is the reciprocal of 120 Hz). Indeed, as shown in Figure 3C, the theoretical distribution (red line) approximates the measurements (blue histogram). The additional 1–3 milliseconds difference between the measured and theoretical value is the result of the additional image processing required to determine if the LED was on. The latency caused by this image processing was not included in the measurements of the first two methods, thus a small amount of additional latency is expected. For a 60 Hz frame rate, the interval is expected to be 5–21.7 milliseconds, and this is also close to the measured values shown in Figure 3D. Finally, with a 30 Hz frame rate, the interval is expected to be 5–38.3 milliseconds, and again this is similar to the measurements (Figure 3E).

Note that all experiments were performed with a very short integration interval – ten microseconds. Longer intervals would directly increase the latency measured above except in the case of the LED-based method (provided that the integration interval remained shorter than the nominal inter-frame interval).

From the measurements above, we can conclude that the image readout and transfer time from the camera to the Motmot software averaged 5 msec with the particular hardware used (Figure 3A–B). As described at the beginning of this section, this includes components outside of Motmot's direct control, such as the particular camera and interface used. The additional latency added by image processing to extract the mean luminance (Figure 3C–E), averages about 1 msec. Although this image processing latency is the only aspect of latency under the direct control of Motmot, it is the cumulative sum of all latencies involved that determines the total system latency.

## FView plugins

The FView application provides a simple plugin mechanism by which users can develop their own realtime image processing alogrithms without worrying about live video display, saving movies, or other functionality provided by FView. The Motmot online and downloadable documentation includes a section on the steps necessary to write a plugin and several tutorial examples. The most important element of the plugin is the user-written process_frame() method. As a consequence of registering the plugin, this method gets called immediately after image acquisition and makes the recently acquired image available for processing. In order to minimize latency, this method is called from the camera acquisition thread. Therefore, care must be taken when sharing data with other threads, such as from the GUI. The return values of this method are used to draw points and line segments on the FView live display, and other outputs may be performed by the plugin itself, such as sending data over the network or saving it to disk.

## FlyMovieFormat

Several goals motivated the development of a new movie file format. These were 1) single pass, low CPU overhead writing of lossless movies for realtime streaming applications. 2) Precise timestamping for correlation with other activities. And 3) a simple format that can be read from Python, C, and MATLAB. These goals were acheived via using a very simple format. After an initial header containing meta-data such as image size and color coding scheme (e.g. monochromatic 8 bits per pixel, YUV422, etc.), repeated chunks of raw image data and timestamp are saved. Because the raw image data from the native camera driver is saved, no additional processing is performed. Thus, streaming of movies from camera to disk will keep the CPU free for other tasks, but it will require lots of disk space. Furthermore, the disk bandwidth required is equivalent to the camera bandwidth (unless the user saves only a region of the images or only saves a fraction of the incoming frames). For the exact file format definition, please see the online or downloadable documentation.

## Applications built on Motmot

### FlyTrax

**Figure 4:**
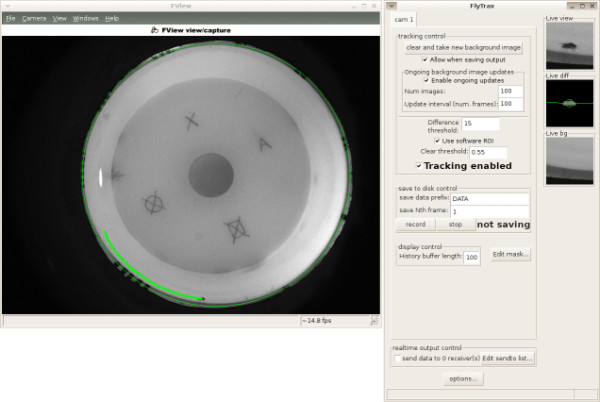
**Screenshot of FView application and FlyTrax plugin**. FView is a relatively simple application which provides live image viewing, camera parameter adjustment, saving of uncompressed movies with accurate timestamp information, and support for plugins. FlyTrax is one such plugin that tracks a single target position and orientation in the 2D view using background subtraction.

FlyTrax is a realtime fly tracking application that tracks the position and orientation of a fly in two dimensions (see Figure 4, for example). Within our laboratory, we have used FlyTrax for tracking the orientation of magnetically tethered flies that were free to rotate about a single axis but were otherwise constrained [6-8]. Additionally, we have used FlyTrax to track flies as they walk freely around planar and three dimensional arenas [9].

The capability to automatically track a fly moving around an arena is a re-implementation of functionality present in other software (e.g. [5,10,11]). Rather than being a purpose-built application, however, FlyTrax is a plugin to FView, and thus illustrates FView's flexibility. Furthermore, the realtime tracking abilities of FlyTrax enables 'virtual open loop' type experiments in which the animals visual feedback loop is artificially broken [5].

Omitting the GUI aspects, a sketch of the operation of the flytrax plugin in pseudocode is below. Note that this is only an approximation of the function calls used. The actual code is more complicated, partly because it utilizes the motmot.realtime_image_analysis module for rapid image analysis.

def process_frame(incoming_image):

update_background_image(incoming_image)

absdiff_image = abs(incoming_image-background_image)

pixel_location = argmax(absdiff_image)

subpixel_location, orientation = process_image_region(incoming_image,pixel_location)

save_to_disk(subpixel_location, orientation)

update_visual_display(subpixel_location, orientation)

def process_image_region(incoming_image,pixel_location):

local_image = incoming_image.extract_small_image_in_neighborhood(pixel_location)

subpixel_location_x = local_image.mean(axis=x)/local_image.sum(axis=x)

subpixel_location_y = local_image.mean(axis=y)/local_image.sum(axis=y)

subpixel_location = (subpixel_location_x, subpixel_location_y)

C = calc_covariance_matrix(local_image)

eigenvalues, eigenvectors = calc_eigen(C)

max_eigenvalue_idx = argmax(eigenvalue)

orientation = eigenvectors [max_eigenvalue_idx]

return subpixel_location, orientation

Another plugin, called 'trackem', is similar to FlyTrax in that it tracks points in realtime. The main difference is that it tracks multiple points simultatneously, detecting only the darkest or lightest points without using background subtraction. This plugin has been used to track a remotely controlled helicopter and estimate its pose with sufficiently low latency and high accuracy to enable a computer to perform the control [12].

### External applications

For the purposes of illustration, two additional applications based on motmot are described here, but not included with Motmot. 'Flydra' is a realtime multi-camera 3D tracking system for flying animals [13] and [**Multi-camera Realtime 3D Tracking of Multiple Flying Animals**. *Computer Vision and Image Understanding *In review.]

This system allows the tracking of the position and orientation of freely moving animals with minimal latency using arbitrary numbers of cameras (eleven have been tested). As such, it allows virtual open loop experiments to be performed on freely moving animals in large experimental spaces [14]. Flydra operates by having dedicated image processing computers, each of which extract and transfer a small amount of information about the detected animals' image location back to a central computer on the arrival of each frame. The central computer is also connected to the CamTrig USB device and thus coordinates the timing of all incoming image frames. Flydra is built on Motmot, utilizing all components described in Figure 1 with the exception of FView and its plugins, which are replaced with a command-line program with no GUI that does image processing using the motmot.realtime_image_analysis and motmot.FastImage packages. FView itself is not used within Flydra for two reasons. First, Flydra runs on computers distributed throughout a network, and the graphical interface of FView is a hindrance for this type of operation. Second, FView currently supports only a single camera per running instance, and Flydra often runs more than one camera per computer.

'CTrax' is an offline image analysis program that tracks multiple flies walking in an arena, while maintaining the identity of individuals over minutes to hours [15]. In addition to using motmot.FlyMovieFormat as a supported movie input format, CTrax uses several software components such as motmot.wxglvideo to implement GUI tasks such as rapid display of video data.

## Competing interests

The authors declare that they have no competing interests.

## Authors' contributions

ADS wrote the software and the manuscript. MHD supervised the project and preparation of the manuscript. Both authors read and approved the final manuscript.
